# Plasma amino acid concentrations during experimental hyperinsulinemia in 2 laminitis models

**DOI:** 10.1111/jvim.16095

**Published:** 2021-03-11

**Authors:** Simon M. Stokes, Darko Stefanovski, François‐René Bertin, Carlos E. Medina‐Torres, James K. Belknap, Andrew W. van Eps

**Affiliations:** ^1^ Australian Equine Laminitis Research Unit, School of Veterinary Science The University of Queensland Gatton Queensland Australia; ^2^ Department of Clinical Studies, School of Veterinary Medicine New Bolton Center, University of Pennsylvania Kennett Square Pennsylvania USA; ^3^ Department of Veterinary Clinical Sciences, College of Veterinary Medicine The Ohio State University Columbus Ohio USA

**Keywords:** amino acids, endocrinopathic, horse, hypoaminoacidemia, insulin

## Abstract

**Background:**

Endocrinopathic laminitis develops in association with insulin dysregulation, but the role of insulin in the pathogenesis remains unclear. Hyperinsulinemia can cause hypoaminoacidemia, which is associated with integumentary lesions in other species and therefore warrants investigation as a potential mechanism in laminitis.

**Objective:**

Evaluate plasma amino acid concentrations in the euglycemic‐hyperinsulinemic clamp (EHC) and prolonged glucose infusion (PGI) laminitis models.

**Animals:**

Sixteen Standardbred horses.

**Methods:**

Prospective experimental study. Plasma amino acid concentrations were measured in samples collected every 6 hours from horses that underwent a 48‐hour EHC (n = 8) or 66‐hour PGI (n = 8) after a 24‐ or 6‐hour baseline period in EHC and PGI groups, respectively.

**Results:**

Fifteen of the 20 measured amino acid concentrations decreased over time in both EHC and PGI horses (*P* < 0.001). The median percentage change from baseline for these amino acids was: histidine (EHC: 41.5%; PGI: 43.9%), glutamine (EHC: 51.8%; PGI: 35.3%), arginine (EHC: 51.4%; PGI: 41%), glutamic acid (EHC: 52.4%; PGI: 31.7%), threonine (EHC: 62.8%; PGI: 25.2%), alanine (EHC: 48.9%; PGI: 19.5%), proline (EHC: 56.2%; PGI: 30.3%), cystine (EHC: 34.9%; PGI: 31.2%), lysine (EHC: 46.4%; PGI: 27.8%), tyrosine (EHC: 27.5%; PGI: 16.9%), methionine (EHC: 69.3%; PGI: 50.8%), valine (EHC: 50.8%; PGI: 34.4%), isoleucine (EHC: 60.8%; PGI: 38.7%), leucine (EHC: 48.2%; PGI: 36.6%), and phenylalanine (EHC: 16.6%; PGI: 12.1%).

**Conclusions and Clinical Importance:**

Hypoaminoacidemia develops in EHC and PGI laminitis models. The role of hypoaminoacidemia in the development of hyperinsulinemia‐associated laminitis warrants further investigation.

**Abbreviations**
EHC: euglycemic‐hyperinsulinemic clamp
IGF‐1R: insulin‐like growth factor‐1 receptor
NME: necrolytic migratory erythema
PGI: prolonged glucose infusion
SND: superficial necrolytic dermatitis
UPLC: ultra performance liquid chromatography

## 1 INTRODUCTION

Most clinical cases of laminitis in horses develop in association with insulin dysregulation, a key component of equine metabolic syndrome and a common finding in horses diagnosed with pituitary pars intermedia dysfunction.^1^, ^2^, ^3^, ^4^ Prior research has shown that horses with hyperinsulinemia are at high risk of developing laminitis and that hyperinsulinemia induces laminitis in horses undergoing a prolonged euglycemic‐hyperinsulinemic clamp (EHC) or a prolonged glucose infusion (PGI).^5^, ^6^, ^7^, ^8^, ^9^ Although these findings indicate that hyperinsulinemia plays an important role in the pathogenesis of endocrinopathic laminitis, the mechanism by which hyperinsulinemia induces laminitis remains unclear. Results of previous studies have supported the hypothesis that insulin exerts direct effects within the lamellar tissue via the insulin‐like growth factor‐1 receptor (IGF‐1R).^10^, ^11^, ^12^ Results from recent research however question whether insulin (at physiological concentrations) is capable of binding lamellar IGF‐1 receptors,^13^ and limited evidence supports that insulin itself directly causes local lamellar pathology by other mechanisms. Therefore, studies aimed at investigating the systemic effects of hyperinsulinemia during the development of laminitis may lead to improved understanding of the key mechanisms responsible for the development of lamellar pathology.

Insulin is a major regulator of protein metabolism, with hyperinsulinemia having been shown to decrease plasma amino acid concentration by altering amino acid utilization and protein turnover in several mammalian tissues including skeletal muscle and skin.^14^, ^15^, ^16^, ^17^, ^18^ Importantly, hyperinsulinemia‐induced hypoaminoacidemia previously has been reported in horses undergoing a short‐term (2 hour) EHC.^15^ This finding may have relevance to laminitis because hypoaminoacidemia causes cutaneous pathology in humans and dogs, which resolves after therapeutic correction of blood amino acid concentrations.^19^, ^20^, ^21^ In addition, amino acid deprivation has been shown to affect cell adhesion and cytoskeletal dynamics in human cells.^22^, ^23^ Lamellar pathology associated with hyperinsulinemia including loss of lamellar epithelial cell adhesion, cell stretch, and extracellular matrix degradation^5^, ^24^, ^25^ are consistent with altered turnover of lamellar structural proteins (eg, cytoskeletal proteins, adhesion molecules, and extracellular matrix proteins) because of low amino acid concentrations.

The EHC and PGI laminitis models both induce prolonged hyperinsulinemia. An important difference however between the 2 models is that supraphysiological hyperinsulinemia is induced in the EHC model using exogenous insulin, whereas the endogenous hyperinsulinemia induced in the PGI model (by an IV glucose infusion) is comparable to that observed in naturally occurring cases of hyperinsulinemia‐associated laminitis.^1^ Plasma amino acid concentrations have not been investigated during the developmental phase of laminitis in the EHC and PGI laminitis models of endocrinopathic laminitis. We hypothesized that hyperinsulinemia would cause persistent hypoaminoacidemia during the developmental phase of laminitis in the EHC and PGI laminitis models. To investigate this hypothesis, plasma concentrations of the common 20 amino acids were quantified using ultra performance liquid chromatography (UPLC) during the developmental phase of laminitis in the EHC and PGI laminitis models.

## 2 MATERIALS AND METHODS

### 2.1 Animal protocol

Plasma samples from healthy Standardbred horses, which underwent a 48‐hour EHC (n = 8 [8 geldings], mean age, 6.3 ± 1.7 years; mean body weight, 447.8 ± 36.9 kg) or 66‐hour PGI (n = 8 [6 geldings, 2 mares]; mean age, 6.4 ± 1.1 years; mean body weight, 419.9 ± 13.9 kg) were used. All horses were from the same population and were recently retired (<4 weeks) from racing. All horses were sound at the walk and had no gross or radiographic abnormalities of the feet. For the duration of the experiment, horses were confined to stocks (EHC horses) or cross‐tied in a stall (PGI horses) and received ad libitum access to alfalfa hay and water.

An EHC was performed for 48 hours as previously described^5^ after a 24‐hour period without intervention (total study period, 72 hours). Briefly, an IV bolus (45 mIU/kg) of recombinant human insulin (Humulin‐R, Eli‐lily, West Ryde, Australia) diluted in 50 mL of 0.9% sodium chloride (Baxter, Old Toongabbie, Australia) was administered via a 14‐gauge catheter placed in the right jugular vein and immediately was followed by a continuous IV infusion of insulin in 0.9% sodium chloride (Baxter) at a rate of 6 mIU/kg/min. A continuous IV infusion of 50% glucose (Baxter) was administered concurrently via the right jugular catheter, with the administration rate adjusted to maintain euglycemia (4.0 ± 1.0 mmol/L).

A PGI was performed for 66 hours as previously described^7^ after a 6‐hour period without intervention (total study period, 72 hours). Briefly, a continuous IV infusion of 50% glucose (Baxter) was administered via a 14‐gauge catheter placed in the right jugular vein at a rate of 0.68 mL/kg/h. Blood glucose concentration was measured using a portable glucometer (Roche Diagnostics, Indianapolis, Indiana) at 0 hours, 15 minutes, 30 minutes, 1 hour, 90 minutes, 100 minutes, 110 minutes, 2 hours, then hourly until 6 hours, and then every 6 hours for the remainder of the experiment. The glucose infusion rate was decreased in 10% decrements if the blood glucose concentration exceeded 15 mmol/L.

For the duration of the experiments, horses were monitored constantly and vital signs were measured every 2 hours. After the study period, the horses were euthanized using pentobarbital sodium (20 mg/kg IV).

### 2.2 Measurement of plasma free amino acids

Blood for plasma amino acid determinations was withdrawn from the left jugular vein via a 14‐gauge catheter (designated for blood sampling) after discarding 20 mL of blood. Samples were collected into evacuated tubes containing lithium heparin (Becton Dickinson, Franklin Lakes, New Jersey) and were taken during the pre‐EHC or pre‐PGI (BASELINE) period (EHC horses: 0 hours, 12 hours, and 24 hours; PGI horses: 0 hours and 6 hours) and during the laminitis induction period (EHC horses: 30 hours, 36 hours, 48 hours, 54 hours, and 66 hours; PGI horses: 12 hours, 18 hours, 30 hours, 36 hours, 48 hours, and 72 hours). After blood collection, the catheter was irrigated with 5 mL of 0.9% sodium chloride (Baxter). Samples were immediately centrifuged and plasma stored at −80°C.

The plasma concentrations of amino acids were determined using precolumn derivatization amino acid liquid chromatography with 6‐aminoquinolyl‐N‐hydroxysuccinimidyl carbamate followed by separation of the derivatives and quantification by modified reversed phase UPLC. The column employed was a Waters Acquity UPLC BEH C18 1.7 μm × 100 mm column (Waters Corporation, Milford, Massachusetts) with detection at 260 nm (ultraviolet) and a flow rate of 0.7 mL/min. This approach enabled a 12‐minute analysis time per sample. Briefly, plasma stored at −80°C was thawed to room temperature and then diluted 1 : 1 with 200 μM of an internal standard (DL‐Norvaline, Sigma‐Aldrich, St. Louis, Missouri). The solution was deproteinated by ultrafiltration through a 10 kDa molecular weight cutoff spin filter (Amicon Ultra Centrifugal Filters, Merck, Darmstadt, Germany) at 4800*g* for 60 minutes at 5°C. Twenty μL of filtrate then was derivatized using the AccQ‐Tag Ultra Derivatization Kit (Waters Corporation) following the supplier's recommended procedures. Standards for detection and quantitation of amino acids were prepared using Amino Acid Standard H (Thermo Fisher Scientific, Waltham, Massachusetts) with the addition of asparagine, glutamine, and tryptophan with DL‐Norvaline (Sigma‐Aldrich) as the internal standard. Quantitation of the amino acids was performed using Waters Empower2 software (Waters Corporation). The data are reported as concentration (μg/mL). Percentage change values were calculated by finding the median of: ([last EHC or PGI measurement − first baseline measurement]/first baseline measurement) × 100.

### 2.3 Measurement of serum insulin in PGI horses

Blood was withdrawn from the left jugular vein via a 14‐gauge catheter (designated for blood sampling) after discarding 20 mL of blood. Samples were collected into evacuated silicate‐containing serum tubes (Becton Dickinson) every 6 hours. Samples were immediately centrifuged and serum was stored at −80°C. Serum samples were analyzed using a commercially available chemiluminescence immunoassay (Immulite 1000, Chemiluminescent Assay, Siemens, Bayswater, Australia) to determine serum immunoreactive insulin concentrations. Measurements are presented as μIU/mL. Serum insulin concentrations were not determined in the EHC horses because serum insulin concentrations previously have been reported in the same population of horses undergoing the same EHC protocol.^6^

### 2.4 Data analysis

All analyses were conducted using Stata 15.1MP (StataCorp, College Station, Texas), with 2‐sided tests of hypotheses and *P <* .05 as the criterion for statistical significance. Age and weight data were analyzed for normality using Shapiro‐Wilk tests. Descriptive statistics are expressed as mean with SD for normally distributed variables and median (interquartile range [IQR]) for skewed data and tabulation of categorical variables. Inference statistical analysis was based on a multilevel mixed‐effects linear regression model with 2 fixed effects: continuous time and group (EHC vs PGI). Random effects were set on the level of the animal. Robust estimation of the variance was used to permit departures from the normality of the outcome data. Estimates produced with the robust estimator are not subject to the assumption of homogeneity of the variance and are different for each of the coefficients of the model. The slopes of the linear trends for each analyte were generated by the model and analyzed to determine if there was a change over time in each group followed by a comparison of these slopes between groups (EHC vs PGI) to determine if there was an effect of treatment. Post hoc analysis was performed on the model‐adjusted means. The least significant difference method was used to adjust the significance values for multiple pairwise comparisons.

## 3 RESULTS

### 3.1 Clinical variables

All EHC horses developed incessant weight shifting (Obel grade 1 laminitis) 28‐34 hours after initiating the EHC and histological lesions including severe secondary epidermal lamellae elongation with dermo‐epidermal separation compatible with lamellar failure. A detailed description of the lamellar histopathology in EHC horses has been published.^5^

The PGI horses developed increased digital pulse amplitude and the feet were warm to the touch from 24 hours onward. Sixty‐six hours after initiating the PGI, 3 horses were not detectably lame and 5 had mild lameness noted at the walk particularly on turning (Obel grade 2 laminitis). The PGI horses developed mild lamellar histopathology (data not shown) compatible with laminitis, similar to that previously reported in Standardbred horses from the same population that underwent the PGI laminitis model.^7^ Serum insulin concentrations for PGI horses are presented in Figure S1. The median (IQR) serum insulin concentration during the PGI was 132.9 μIU/mL (94.2‐152.8) and the highest insulin concentration was observed at 54 hours (183 μIU/mL [60.7‐226]). The mean ± SEM serum insulin concentration of Standardbred horses from the same population that underwent the same EHC protocol has been reported previously as 1036 μIU/mL ± 129.^6^

### 3.2 Plasma free amino acids

**FIGURE 1:**
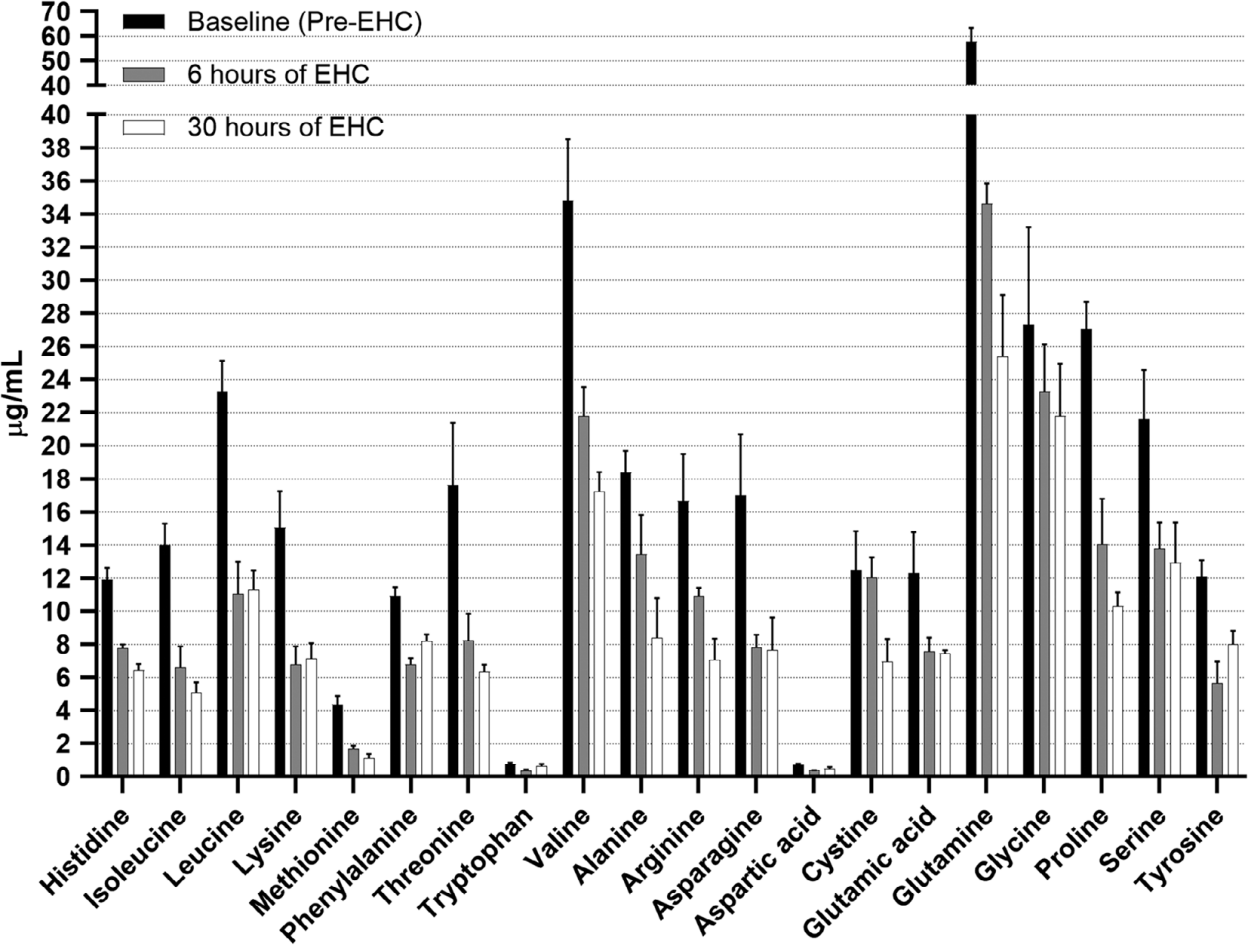
Median (IQR) plasma amino acid concentrations in horses undergoing a 48 hour EHC after a 24 hour baseline period without intervention. Time points presented are baseline (pre‐EHC) and 6 hours and 30 hours after commencing the EHC. All amino acids decreased over time (*P <* .001) except aspartic acid (*P =* .3). EHC, euglycemic‐hyperinsulinemic clamp

**FIGURE 2:**
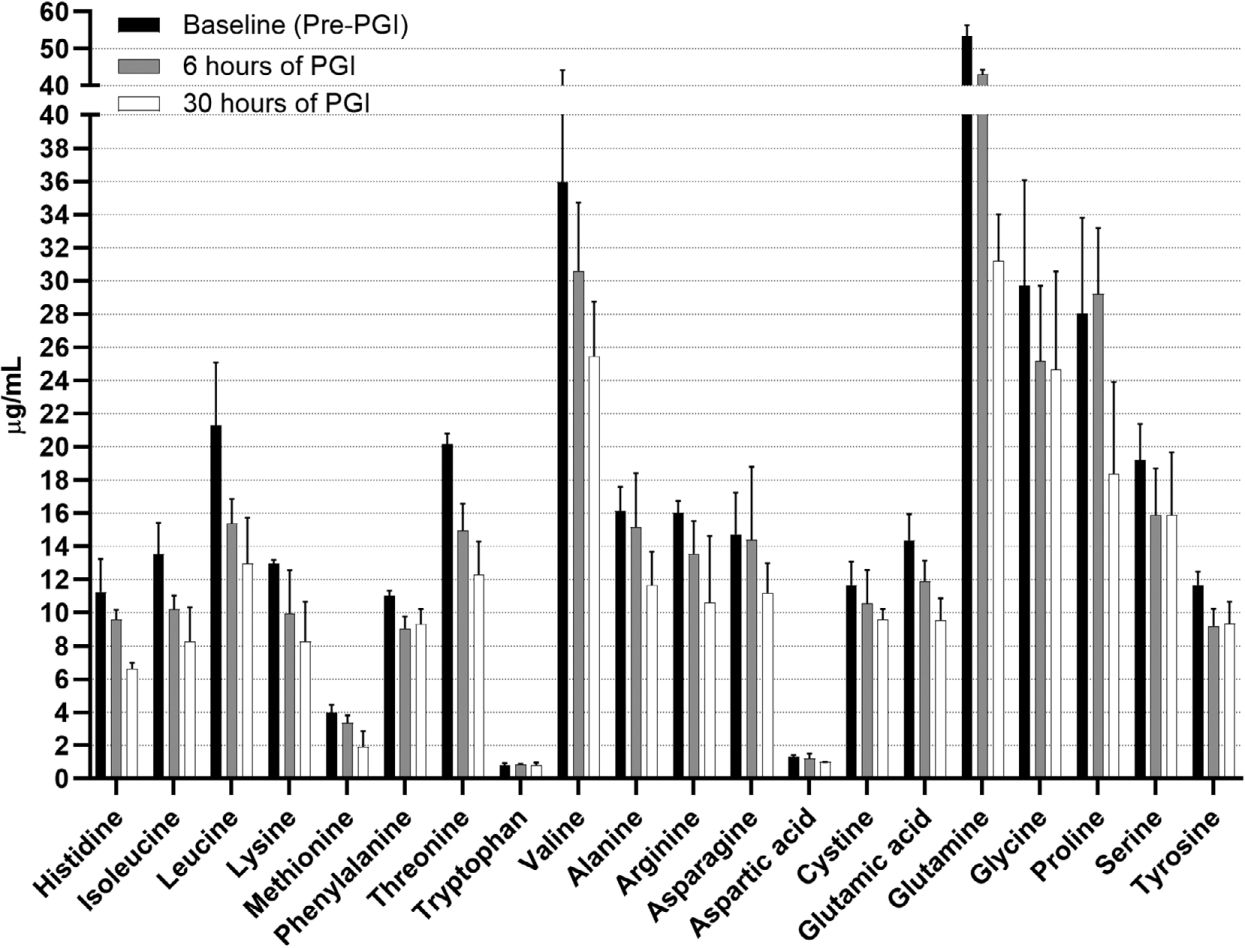
Median (IQR) plasma amino acid concentrations in horses undergoing a 66 hour PGI after a 6 hour baseline period without intervention. Time points presented are baseline (pre‐PGI) and 6 hours and 30 hours after commencing the PGI. All amino acids decreased over time (*P <* .05) except asparagine (*P =* .09), serine (*P =* .1), and tryptophan (*P =* .6). PGI, prolonged glucose infusion

The majority of the common 20 amino acids (EHC, 19/20; PGI, 15/20) decreased rapidly and markedly below baseline concentrations within 6 hours of commencing the EHC and PGI, and remained below baseline for the duration of the EHC and PGI (Figures 1 and 2, Figures S2 and S3). The most marked decreases occurred in the concentrations of asparagine, glutamine, aspartic acid, threonine, alanine, proline, lysine, tyrosine, methionine, valine, isoleucine, and leucine, which decreased to below 50% of baseline in the EHC group. In the PGI group, histidine, glutamine, aspartic acid, threonine, methionine, valine, isoleucine, and leucine decreased to below 65% of baseline.

#### 3.2.1 Essential amino acids

**FIGURE 3:**
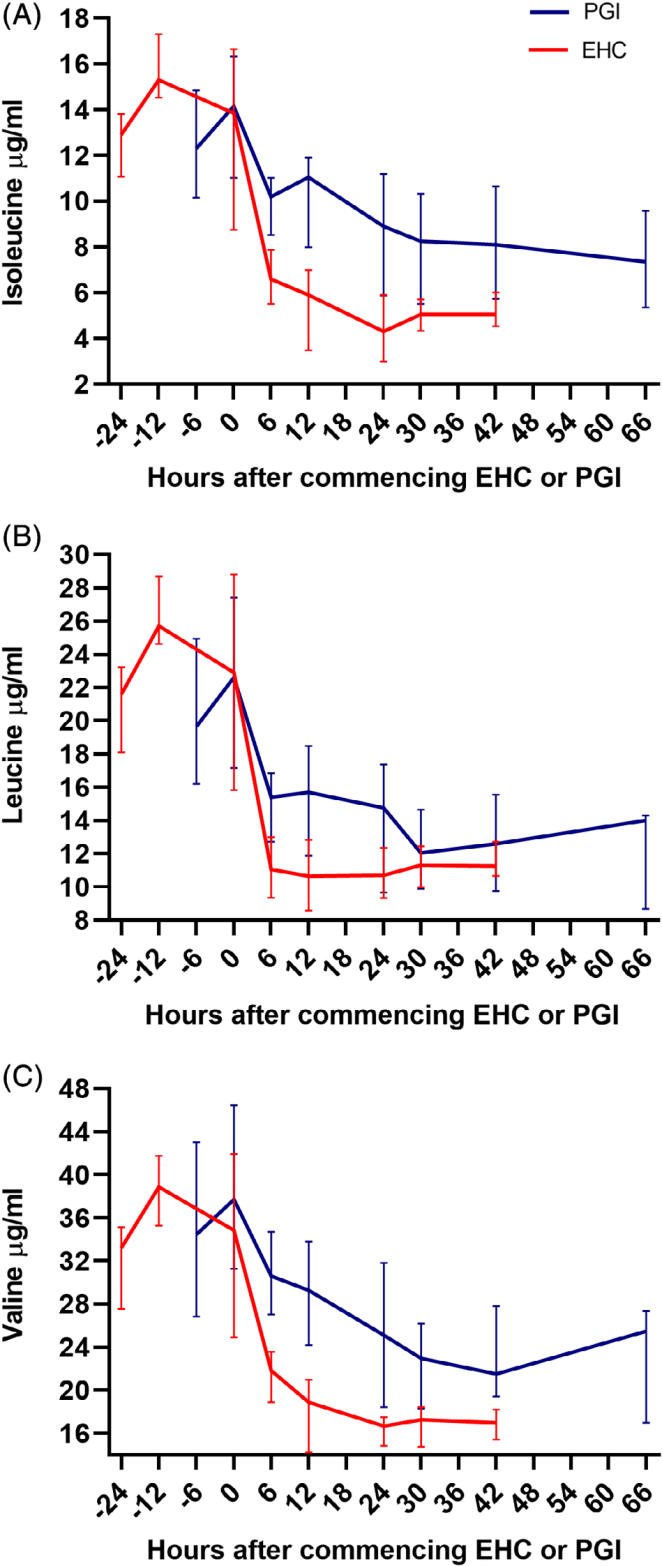
Median (IQR) plasma branched chain amino acid concentrations in horses undergoing a 48 hour EHC or 66 hour PGI after a 24 hour or 6 hour baseline period in EHC and PGI groups, respectively. Leucine (A), isoleucine (B), and valine (C) decreased over time in EHC and PGI horses (*P <* .001) and there was a relative decrease in leucine, isoleucine, and valine in EHC horses compared to PGI horses over time (*P <* .001). EHC, euglycemic‐hyperinsulinemic clamp; PGI, prolonged glucose infusion

Histidine, threonine, lysine, methionine, isoleucine, leucine, valine, and phenylalanine decreased over time in the EHC (Figures 1and 3) and PGI (Figures 2 and 3) groups (*P <* .001). Tryptophan decreased over time in EHC (*P <* .001) but not in PGI (*P* ≥ .05). There was a relative decrease in threonine, lysine, methionine, isoleucine, leucine, valine, and phenylalanine concentrations in EHC compared to PGI over time (*P <* .05). There was no change in histidine and tryptophan concentrations in EHC compared to PGI over time (*P* = .05).

The median (IQR) percentage change from baseline was: histidine (EHC: 41.5% [33.3‐58.9]; PGI: 43.9% [39.1‐58.2]), threonine (EHC: 62.8% [44.7‐66.7]; PGI: 25.2% [1.3‐37.4]), lysine (EHC: 46.4% [36.6‐59.3]; PGI: 27.8% [4.2‐41.5]), methionine (EHC: 69.3% [59.2‐80.2]; PGI: 50.8% [24.9‐64.8]), valine (EHC: 50.8% [32.7‐55.9]; PGI: 34.4% [18.5‐40.6]), isoleucine (EHC: 60.8% [46.2‐66.8]; PGI: 38.7% [30.6‐51.3]), leucine (EHC: 48.2% [27.3‐55.4]; PGI: 36.6% [18.1‐43.6]), phenylalanine (EHC: 16.5% [9.1‐24.4]; PGI: 12.1% [6.6‐33.9]), and tryptophan (EHC: 21.9% [11.6‐44.5]; PGI: 24.1% [4.2‐40.5]).

#### 3.2.2 Nonessential amino acids

Asparagine, serine, glutamine, arginine, glycine, glutamic acid, alanine, proline, cystine, and tyrosine decreased over time in the EHC group (*P <* .001; Figure 1). Aspartic acid did not change over time in EHC (*P* = .3).

Glutamine, arginine, glycine, aspartic acid, glutamic acid, alanine, proline, cystine, and tyrosine decreased over time in PGI (*P <* .001) (Figure 2). Asparagine did not change over time in PGI (*P =* .1). There was a relative decrease in asparagine, serine, glutamine, arginine, aspartic acid, alanine, proline, and cystine in EHC compared to PGI over time (*P <* .05). There was no change in glycine or glutamic acid concentrations in EHC compared to PGI over time (*P* > .05).

The median (IQR) percentage change from baseline was: asparagine (EHC: 65.6% [33.3‐73.3]; PGI: 36.2% [23.4‐74.9]), serine (EHC: 31.5% [21.9‐46.9]; PGI: 44.9% [14.1‐86.9]), glutamine (EHC: 51.9% [41.7‐59.2]; PGI: 35.3% [11.4‐38.5]), arginine (EHC: 51.5% [37.5‐54.9]; PGI: 41% [29.5‐66.5]), glycine (EHC: 29.3% [14.6‐43.2]; PGI: 39.5% [22.1‐58]), aspartic acid (EHC: 66.5% [29.4‐75.6]; PGI: 39.4% [22.9‐58.7]), glutamic acid (EHC: 52.4% [44.5‐58.1]; PGI: 31.7% [27.9‐39.1]), alanine (EHC: 48.9% [28.3‐58.6]; PGI: 19.5% [1.7‐63.5]), proline (EHC: 56.2% [25.5‐62.4]; PGI: 30.3% [11.6‐37.6]), cystine (EHC: 34.9% [15.7‐51]; PGI: 31.2% [20.5‐40.3]), tyrosine (EHC: 27.5% [11.8‐33.7]; PGI: 16.9% [5.9‐48]).

## 4 DISCUSSION

Marked and sustained hypoaminoacidemia developed rapidly with both the EHC (exogenous hyperinsulinemia) and the PGI (endogenous hyperinsulinemia). Hypoaminoacidemia also has been reported in humans, goats, and pigs undergoing an EHC,^14^, ^26^, ^27^, ^28^, ^29^ and additional studies have characterized the effects of insulin on protein metabolism.^30^, ^31^ Current research indicates that insulin‐induced hypoaminoacidemia occurs as a result of decreased whole body proteolysis and oxidation of essential amino acids,^26^, ^27^ but the mechanisms by which insulin regulates protein turnover (ie, protein synthesis and proteolysis) is complex and not completely understood. Furthermore, plasma amino acid concentrations have been shown to influence the effects of hyperinsulinemia on protein metabolism, with hyperinsulinemia having been shown to stimulate protein synthesis in the presence of increased plasma amino acid concentrations.^32^, ^33^ In contrast, hypoaminoacidemia results in decreased rates of protein synthesis and blunts the responsiveness of insulin's suppression of proteolysis.^29^ In a study of healthy horses undergoing EHC for 2 hours, it was found that hyperinsulinemia increased whole body and muscle protein synthesis.^15^ In our study, prolonged hyperinsulinemia (ie, >2 hours) resulted in sustained hypoaminoacidemia in both the EHC and PGI horses, which suggests that although protein synthesis rates initially may increase in response to hyperinsulinemia in the EHC and PGI horses, it is likely that protein synthesis rates would have been limited after the development of hypoaminoacidemia, observed as soon as 6 hours after commencing EHC and PGI. Importantly, the decrease in amino acid concentrations observed in our study indicates that an altered rate of whole body protein turnover occurs during the developmental phase of laminitis in horses undergoing EHC and PGI laminitis models.

Relevant to the development of hyperinsulinemia‐associated laminitis, hypoaminoacidemia potentially could alter lamellar structural protein turnover, particularly the synthesis and breakdown of lamellar cytoskeletal proteins, cell adhesion molecules and extracellular matrix proteins resulting in the development of lamellar pathology. In epithelial tissues, structural proteins are constantly synthesized and degraded in order to maintain tissue function.^34^ In contrast to other epithelial tissues (eg, skin), the lamellar tissue is unique in that it must withstand the extreme mechanical stresses of weight‐bearing.^35^ Therefore, the structural integrity of the lamellar tissue is potentially highly dependent on adequate plasma amino acid supply in order to maintain lamellar structural protein homeostasis. In our study, marked hypoaminoacidemia occurred shortly after the development of hyperinsulinemia in EHC and PGI horses, which suggests that an amino acid limitation potentially could contribute to the development of laminitis by an inadequate supply of amino acids required for the synthesis of lamellar structural proteins. In support of this concept, lamellar epithelial cell stretch, loss of epithelial cell adhesion, and decreased density of lamellar epithelial cell adhesion molecules (hemidesmosomes) has been reported in the EHC model,^5^, ^24^, ^36^ with similar pathology reported in the PGI model.^7^ Furthermore, supporting a role for hypoaminoacidemia in the development of lamellar pathology, the severity of hypoaminoacidemia was higher in the EHC horses relative to PGI horses, and more severe lamellar histopathology has been reported in the EHC model relative to the PGI model of laminitis.^5^, ^7^ Indeed, lamellar tissue amino acid concentrations and lamellar structural protein metabolism were not evaluated in our study and these factors warrant further investigation.

The endogenous hyperinsulinemia observed in the PGI horses of our study is comparable to that observed in naturally occurring cases of hyperinsulinemia‐associated laminitis.^1^ Importantly, this degree of endogenous hyperinsulinemia rapidly induced marked hypoaminoacidemia, which supports that insulin‐induced hypoaminoacidemia could contribute to the development of naturally occurring laminitis, and indeed is consistent with the insidious nature of hyperinsulinemia‐associated laminitis. Of note, the severity of hyperinsulinemia in the PGI horses (median [IQR], 132.9 μIU/mL [94.2‐152.8]) was less than that previously reported (mean ± SEM; 208 ± 26.1 μIU/mL) in Standardbred horses that underwent the same model.^7^ This discrepancy can be explained by the fact that different immunoassays were used to measure serum insulin concentrations.^37^

The branched‐chain amino acids (leucine, isoleucine, and valine) have been shown to be markedly decreased in humans undergoing an EHC.^14^, ^26^ In our study, all branched‐chain amino acids decreased during the developmental phase of laminitis, with leucine, isoleucine, and valine concentrations observed to be below baseline 6 hours after commencing EHC and PGI. Importantly, deficiencies of branched‐chain amino acids (particularly isoleucine) are associated with the development of severe skin lesions in humans including erythematous plaques, parakeratosis, and inflammatory cell infiltrate, which resolves after supplementation of the deficient amino acid.^21^, ^38^, ^39^, ^40^ Although the exact mechanisms by which low amino acid concentrations cause skin pathology remains unclear, prior research has shown that isoleucine is essential for normal growth and differentiation of keratinocytes and causes keratinocyte growth arrest when depleted in culture.^38^, ^41^ The marked decrease in branched‐chain amino acids observed in our study during the developmental phase of laminitis suggests that low branched‐chain amino acid concentrations could play a role in causing lamellar pathology and warrants further investigation. Indeed, local lamellar amino acid concentrations could be investigated using a lamellar microdialysis system.^42^

In addition to branched‐chain amino acid deficiencies, deficiencies of other essential and nonessential amino acids are associated with the development of severe skin lesions, with hypoaminoacidemia playing an important role in the development of necrolytic migratory erythema (NME) and superficial necrolytic dermatitis (SND) in humans and dogs, respectively.^20^, ^43^ Importantly, histopathology observed in NME and SND including necrosis, inflammatory cell infiltrate, basal cell proliferation, and keratinocyte degeneration^20^, ^44^ also has been observed in the EHC and PGI laminitis models.^5^, ^7^ Current research suggests low plasma amino acid concentrations cause epidermal protein depletion and subsequent development of skin lesions, particularly at high movement or stress areas (eg, feet, oral cavity, pressure points).^20^, ^44^ Although the severity of hypoaminoacidemia associated with skin lesions in prior studies is similar to that observed in the EHC and PGI horses,^20^ macroscopically apparent skin lesions were not observed in our study, which may be attributed to the relatively short duration of hypoaminoacidemia. However, lamellar pathology previously reported in the EHC and PGI laminitis models^5^, ^7^ (without evidence of other macroscopic epithelial pathology) suggests that the lamellar tissue may be particularly susceptible to injury compared with other epithelial tissues because of its weight‐bearing function. Considering this critical role of withstanding mechanical stress, it is likely there is a high requirement for adequate supply of plasma amino acids to the lamellae, and potentially increased susceptibility of the lamellae to even short term hypoaminoacidemia.

To the best of our knowledge, plasma amino acid concentrations have not been determined in naturally occurring cases of hyperinsulinemia‐associated laminitis and this warrants further investigation because the EHC and PGI models may not mimic the pathophysiological events occurring in natural hyperinsulinemia‐associated laminitis. However, our results indicate that marked hypoaminoacidemia occurs in response to hyperinsulinemia early in the developmental phase of laminitis in the EHC and PGI laminitis models and therefore supports further investigations into the systemic effects of hyperinsulinemia on lamellar structural protein metabolism. The hyperinsulinemic euglycemic euaminoacidemic clamp method has been utilized in other species^27^ and may represent a suitable tool to further evaluate the role of hypoaminoacidemia in the development of hyperinsulinemia‐associated laminitis. In addition, hypoaminoacidemia can be a consequence of other conditions that are associated with laminitis such as sepsis^45^, ^46^, ^47^ and ischemia as a result of persistent load,^48^ which would limit amino acid delivery to the lamellae. Therefore, hypoaminoacidemia may warrant further investigation as a potential mechanism in other forms of laminitis.

## CONFLICT OF INTEREST DECLARATION

Authors declare no conflict of interest.

## OFF‐LABEL ANTIMICROBIAL DECLARATION

Authors declare no off‐label use of antimicrobials.

## INSTITUTIONAL ANIMAL CARE AND USE COMMITTEE (IACUC) OR OTHER APPROVAL DECLARATION

Approved by The University of Queensland Animal Ethics Committee (AEC) that monitors compliance with the Animal Welfare Act (2001) and The Code of Practice for the care and use of animals for scientific purposes (current edition). All animals were monitored continuously by the investigators.

## HUMAN ETHICS APPROVAL DECLARATION

Authors declare human ethics approval was not needed for this study.

## Supporting information


**Figure S1** Median (IQR) serum insulin concentrations in Standardbred horses (n = 8) that underwent a prolonged glucose infusion (PGI laminitis model) for 66 hours after a 6 hour baseline period without intervention.

**Figure S2** Median (IQR) plasma essential amino acid concentrations in horses undergoing a 48 hour euglycemic‐hyperinsulinemic clamp (EHC) or 66 hour prolonged glucose infusion (PGI) after a 24 hour or 6 hour baseline period in EHC and PGI groups, respectively.

**Figure S3** Median (IQR) plasma nonessential amino acid concentrations in horses undergoing a 48 hour euglycemic‐hyperinsulinemic clamp (EHC) or 66 hour prolonged glucose infusion (PGI) after a 24 hour or 6 hour baseline period in EHC and PGI groups, respectively.
