# Supplementary material for: Plasma amino acid concentrations during experimental hyperinsulinemia in 2 laminitis models
Source: J Vet Intern Med. 2021 Mar 11;35(3):1589–96. doi: 10.1111/jvim.16095 (PMC8163125; doi:10.1111/jvim.16095)
Supplement: Supplementary file 1 — Figure S1 Median (IQR) serum insulin concentrations in Standardbred horses (n = 8) that underwent a prolonged glucose infusion (PGI laminitis model) for 66 hours after a 6 hour baseline period without intervention. [file JVIM-35-1589-s002.pdf]

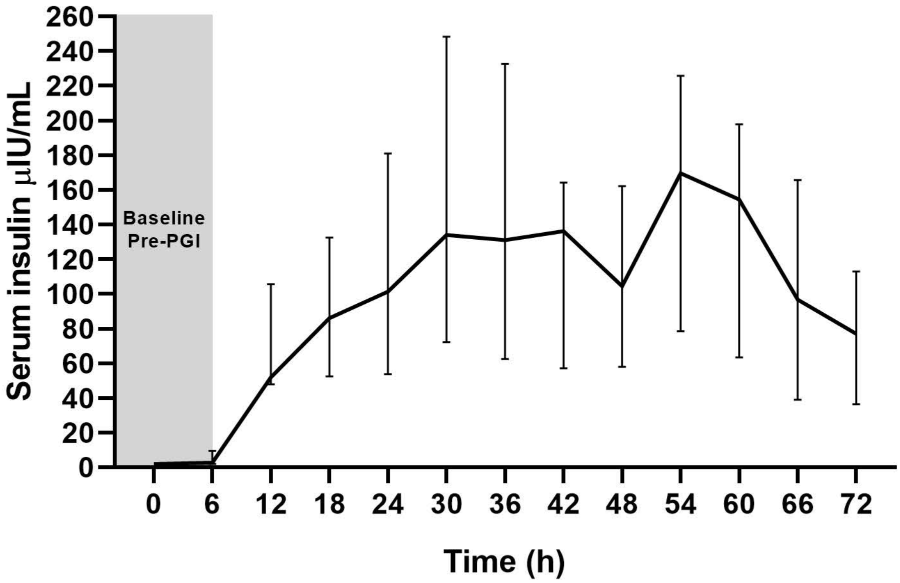

Supplementary Figure 1: Median (IQR) serum insulin concentrations in Standardbred horses (n = 8) that underwent a prolonged glucose infusion (PGI laminitis model) for 66 hours following a 6 hour baseline period without intervention.
