# Supplementary material for: Plasma amino acid concentrations during experimental hyperinsulinemia in 2 laminitis models
Source: J Vet Intern Med. 2021 Mar 11;35(3):1589–96. doi: 10.1111/jvim.16095 (PMC8163125; doi:10.1111/jvim.16095)
Supplement: Supplementary file 3 — Figure S3 Median (IQR) plasma nonessential amino acid concentrations in horses undergoing a 48 hour euglycemic‐hyperinsulinemic clamp (EHC) or 66 hour prolonged glucose infusion (PGI) after a 24 hour or 6 hour baseline period in EHC and PGI groups, respectively. [file JVIM-35-1589-s003.pdf]

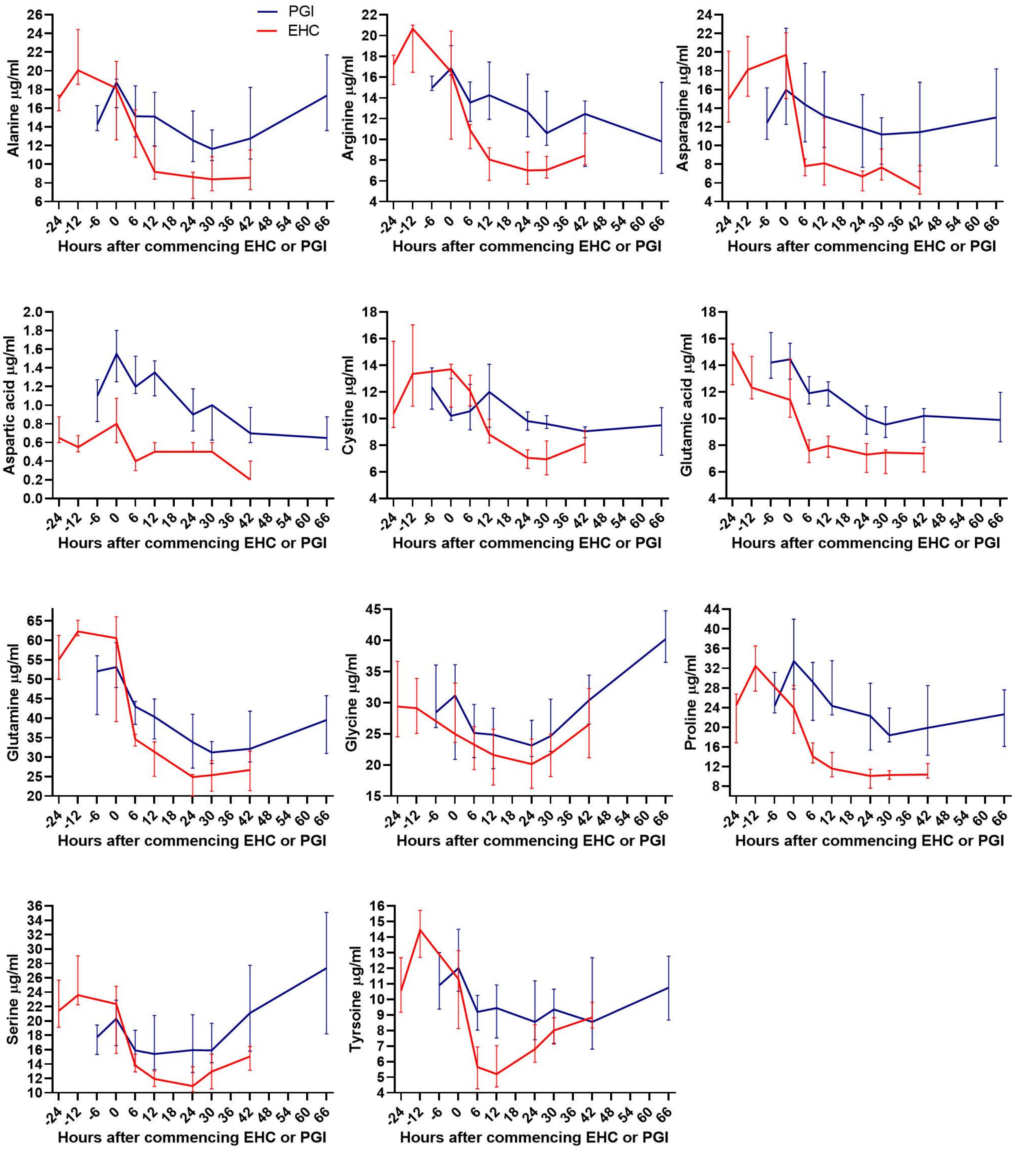

Fig S3: Median (IQR) plasma non-essential amino acid concentrations in horses undergoing a 48 hour euglycemic hyperinsulinemic clamp (EHC) or 66 hour prolonged glucose infusion (PGI) following a 24 hour or 6 hour baseline period in EHC and PGI groups, respectively.
